# An instrument to facilitate value-driven conversations on surveillance technology 

**DOI:** 10.1177/09697330251376894

**Published:** 2025-09-25

**Authors:** Daniëlle van Gaans-Riteco, Annerieke Stoop, Irene Muller-Schoof, Marieke van Vliet, Eveline Wouters

**Affiliations:** Tranzo, Scientific Center for Care and Wellbeing, Tilburg School of Social and Behavioral Sciences Tilburg University, The Netherlands; Groenhuysen, Residential Care Facilities and Care for Older People, Roosendaal, The Netherlands; Tranzo, Scientific Center for Care and Wellbeing, Tilburg School of Social and Behavioral Sciences Tilburg University, The Netherlands; Tranzo, Scientific Center for Care and Wellbeing, Tilburg School of Social and Behavioral Sciences Tilburg University, The Netherlands; Professorship Moral Design Strategy, Fontys University of Applied Science, Eindhoven, The Netherlands; Tranzo, Scientific Center for Care and Wellbeing, Tilburg School of Social and Behavioral Sciences Tilburg University, The Netherlands; School for allied Health Professions, Fontys University of Applied Sciences, Eindhoven, The Netherlands

**Keywords:** perspectives, Schwartz’s values, stakeholders, surveillance technology, value-based implementation (value-based conversation)

## Abstract

**Background:**

The application of care technology is complex, and has an impact on all stakeholders. A specific issue with surveillance technologies is the resulting ethical dilemmas. These dilemmas often touch on people’s values, which arise from their perspectives and determine their attitudes and behaviour. One of the prerequisites for successful stakeholder involvement is knowing and acknowledging their values. Schwartz’s theory of human values has been empirically tested and facilitates the prediction of attitudes and behaviours in different contexts.

**Research aim:**

To develop, test and validate a conversation instrument suitable for use in interviews to explore stakeholders’ values regarding the application of surveillance technologies. The instrument was based on Schwartz’s ten values model and adapted to fit the stakeholders’ (professional) backgrounds, education and language levels.

**Research design:**

We integrated and adapted Schwartz’s ten and nineteen values model, the Personal Value Dictionary, the Portrait Values Questionnaire and Schwartz’s Value Survey to value cards tailored to the stakeholders’ education and language levels. The adaption was an iterative process involving expert consultation. The value cards were validated in 34 interviews with stakeholders involved in the application of surveillance technologies for people with dementia in nursing homes.

**Ethical considerations:**

This study was approved by the Ethical Research Board of Tilburg University (ID TSB_RP771).

**Findings:**

The iterative process resulted in a concept version of the value cards, with expert feedback and ‘member checks’ guiding final revisions. The value cards helped participants articulate their values and were seen as useful tools for reflecting on key considerations related to surveillance technologies.

**Discussion and conclusion:**

Value cards assisted stakeholders in sharing their most important principles regarding the application of surveillance technologies and may help explore their values related to complex technological innovations in the context of psychogeriatrics.

## Introduction

Surveillance technologies often raise legal and ethical dilemmas, influenced by people’s values and determining one’s attitude and behaviour. Given the multitude of stakeholders involved in a nursing home setting, differences in stakeholders’ values, perceptions and interests can be expected with regard to surveillance technologies. Stakeholder involvement and understanding their values is perceived important to contribute to successful application of surveillance technologies. Exploring underlying values is not obvious. Schwartz’ values model^1^ is a widely accepted values model to understand peoples’ values. However, in its current form, this model is not suitable for conducting in-depth conversations with a broad diversity of stakeholders. A format that fits the nursing home stakeholders’ (professional) backgrounds, education and language level is not available yet. Therefore, in a participatory design, this study aimed to develop a conversation instrument based on Schwartz’s values model to explore values of individual stakeholders involved in the application of surveillance technology in nursing homes.

## Background

Every country in the world is expecting an increase in the number and proportion of older people.^1^ As age is the strongest known risk factor for dementia,^1–3^ the number of people with dementia is expected to increase.^4^ The characteristics of dementia result in unpredictable care needs,^5^ associated with concerns about safety and security.^3,6,7^ Dementia has far-reaching physical, psychological and social impacts, not only on people living with the condition but also on their families and care professionals.^8^ Dementia care demands highly targeted and individual approaches due the expansiveness of the condition combined with other health problems that a person might face, which leads to complex challenges in providing effective and sustainable care solutions.^9^ International data indicate that about 30–40% of people diagnosed with dementia will eventually be admitted to a long-term care facility.^7,10–12^ Because people tend to move to nursing homes in the late stages of dementia with complex care needs, these facilities are under high pressure. Combined with shortages of skilled care professionals, this results in societal challenges.^13–15^ One recommendation to address these challenges and make care for older adults with chronic conditions such as dementia sustainable is to foster investment in care technologies.^3,13,16,17^ Technologies such as sensors, remote monitoring systems and GPS technology^18^ could reduce the physical and cognitive strain of nursing staff and informal caregivers^18,19^ by making their work more efficiently and reduce the anxiety about residents’ safety.^18^ In addition, these technologies could compensate for staff shortages without a decrease in quality of care.^20,21^

In this study, we focus on surveillance technologies that allow for visual and auditory monitoring, including tagging and tracking technology, sensors, and audio and video surveillance.^22,23^ Surveillance technologies are increasingly focused on supporting autonomy and respecting privacy while enhancing safety and individualised care for people with dementia.^14,24–26^ Compared to other types of care technology, surveillance technologies such as tagging and tracking technology, sensors, and audio and video surveillance,^22,23^ raises more often legal and ethical dilemmas concerning privacy, safety, freedom of movement and the mental well-being of residents among stakeholders^20.^ Understanding these ethical dimensions calls for recognition that stakeholders may interpret the same concerns differently. For instance, ‘safety’ might mean continuous monitoring to families but maintaining dignity to care staff. Adding to the complexity of dementia care is the degenerative, dynamic, and unpredictable nature of the disease. As dementia progresses, the needs of persons living with dementia, their professional and informal caregivers evolve, and it can be difficult to anticipate how these needs will change.^27^ A participatory approach reveals such nuanced differences and promotes dialogue about underlying values.

Nursing homes focus on a long-term stay in a professional though homelike environment, with an emphasis on well-being rather than care. Several stakeholders live or do (formal, informal or voluntary) work in this environment, in care technology is to be used.^28^ Therefore, care technologies affect several stakeholder groups.^20,29–31^ Beyond the directly involved stakeholders is another group of indirectly involved ones, such as coordinating practitioners, policy officers, managers, information and communication (ICT) employees, developers and surveillance technology vendors.^20,31^ Multiple interdisciplinary stakeholders and interdependencies in healthcare influence the success of care technology implementation.^28^ Moreover, each stakeholder brings a unique background, set of values, perceptions and interests, which may differ from or conflict with others.^32^ This could more easily lead to confusion of roles and conflicts of interest as people’s values determine their attitude and behaviour.^28–31^ Many stakeholders emphasise the importance of being acknowledged and feeling involved and believe that their involvement should be increased.^24,29,30,33^ When stakeholders feel more involved, they are more open to changes and willing to collaborate, which contributes to successful implementations.^24,29,33^ The involvement of all stakeholders and understanding their underlying values is perceived as a prerequisite for the successful application of technology in health care.^34^ A participatory approach helps to understand ethical dimensions by ensuring the acknowledgement of diverse stakeholder perspectives, despite practical challenges in engaging all groups.

**Figure 1.**
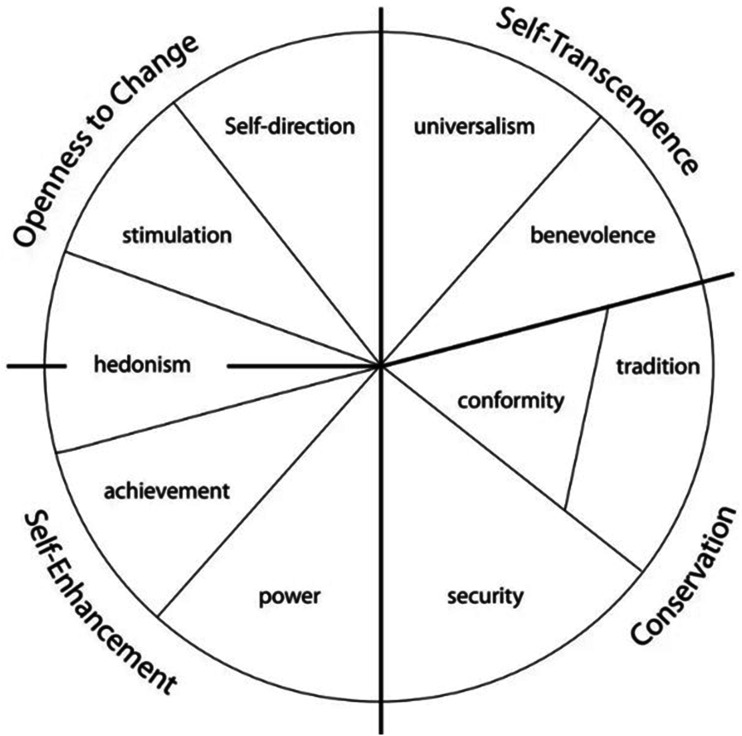
Schwartz’s ten values model.^41^

**Figure 2.**
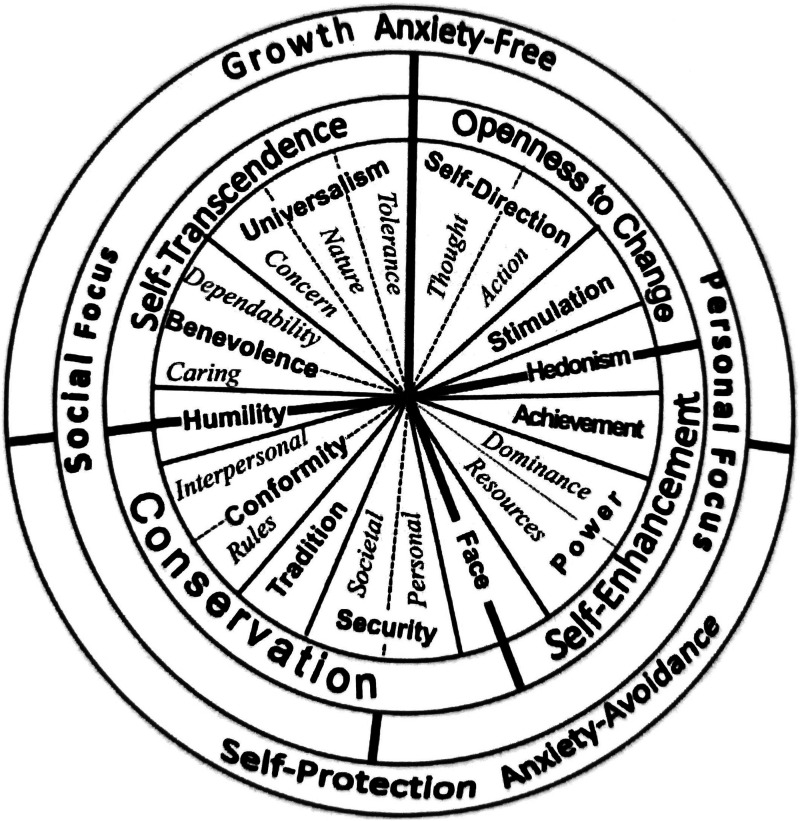
Schwartz’s refined nineteen values model.^39^

Exploring values goes beyond asking people which values they adhere to; they need to be encouraged to express what is important to them.^35^ However, while describing and defining values is complex, analysing them is even more challenging.^36^ Over the years, researchers have conceptualised diverse frameworks to cover nearly all core human values.^37,38^ One of the most widely accepted is Schwartz’s values model,^35^ which is recognised across many cultures and is frequently used to predict attitudes and behaviours in different contexts.^39,40^ Schwartz’s framework, visualised as a circular continuum as shown in Figures 1 and 2, covers the majority of human value judgements^39,41^ and is commonly used by values researchers around the world.^35^ In the past decade, many studies stemming from Schwartz’s human values framework have found systematic relationships between values and various psychological constructs, such as personality traits.^40^ Schwartz’s values model has been applied in psychology,^39,41–43^ sociology,^44^ economics and organisational studies^45,46^ and in studies of individual and collective values.^47,48^ It is also applied to societal challenges such as ethical decision-making, moral authority, and moral strategy formation in municipal and educational issues related to the deployment of smart technologies.^31,49^

While nursing homes involves a broad variety of stakeholders with different backgrounds, educational levels values and interests among which exploring values are of great value, the model of Schwartz is not used in a nursing home setting or in any other similar setting including a wide variety of stakeholders. Moreover, Schwartz’s Value Survey and Portrait Value Questionnaire^50^ assess people’s values through questions scored on a Likert scale. As valuable as this may be for certain settings, exploring values regarding to the application of surveillance technology across a broad diversity of stakeholders in a nursing home, setting requires exploring values in in-depth conversations. The format and the language level of any conversation instrument for exploring stakeholders’ perspectives and values should be suitable and accessible for stakeholders with diverse backgrounds, educational levels and language levels.

Test conversations for this research, starting from Schwartz’s basic human values^39,41^ and subsequently the circular representation of the Moral Design Game,^49^ showed that using the values model in its original (circular) forms to explore stakeholders’ values did not contribute to the quality and clarity of value exploration in interviews. Therefore, we sought a format that better suited the broad diversity of stakeholders in a nursing home setting, both in terms of format and language proficiency. This study aimed to develop a conversation instrument based on Schwartz’s values model to explore a set of values tailored to the diverse range of stakeholders involved in the application of surveillance technology in nursing homes.

## Research methods

The development, testing and validation of a conversational instrument was part of qualitative explorative research in which the perspectives and values of stakeholders, involved in applying surveillance technologies for people with dementia were explored in semi-structured interviews. This article describes how Schwartz’s values were adapted to a format suitable for use in semi-structured explorative in-depth interviews with stakeholders with varying backgrounds, education levels and (Dutch) language levels.

### Development, testing and validation of the conversation instrument

The conversation instrument was created through a participatory, iterative development process. After briefly explaining Schwartz’s theory on basic human values, this section outlines the main activities involved in developing and refining the instrument.

### Schwartz’s theory on basic human values

Schwartz’s basic human values theory specifies a continuum of values based on their motivational congruence ^38^. This framework conceptualises ten related values ordered by importance, forming a system of priorities for groups, societies, and individuals.^41^ Schwartz’s values are categorised into four higher-order values: openness to change, self-enhancement, conservation and self-transcendence. The refined theory of basic human values, which emerged from the ten values model, contains nineteen values.^39^ Table 1 provides an overview of Schwartz’s ten values and nineteen values models. Both value models are also shown in a circular continuum in Figures 1 and 2.

**Table 1.** The four higher-order values, ten basic values and nineteen refined values^50^.

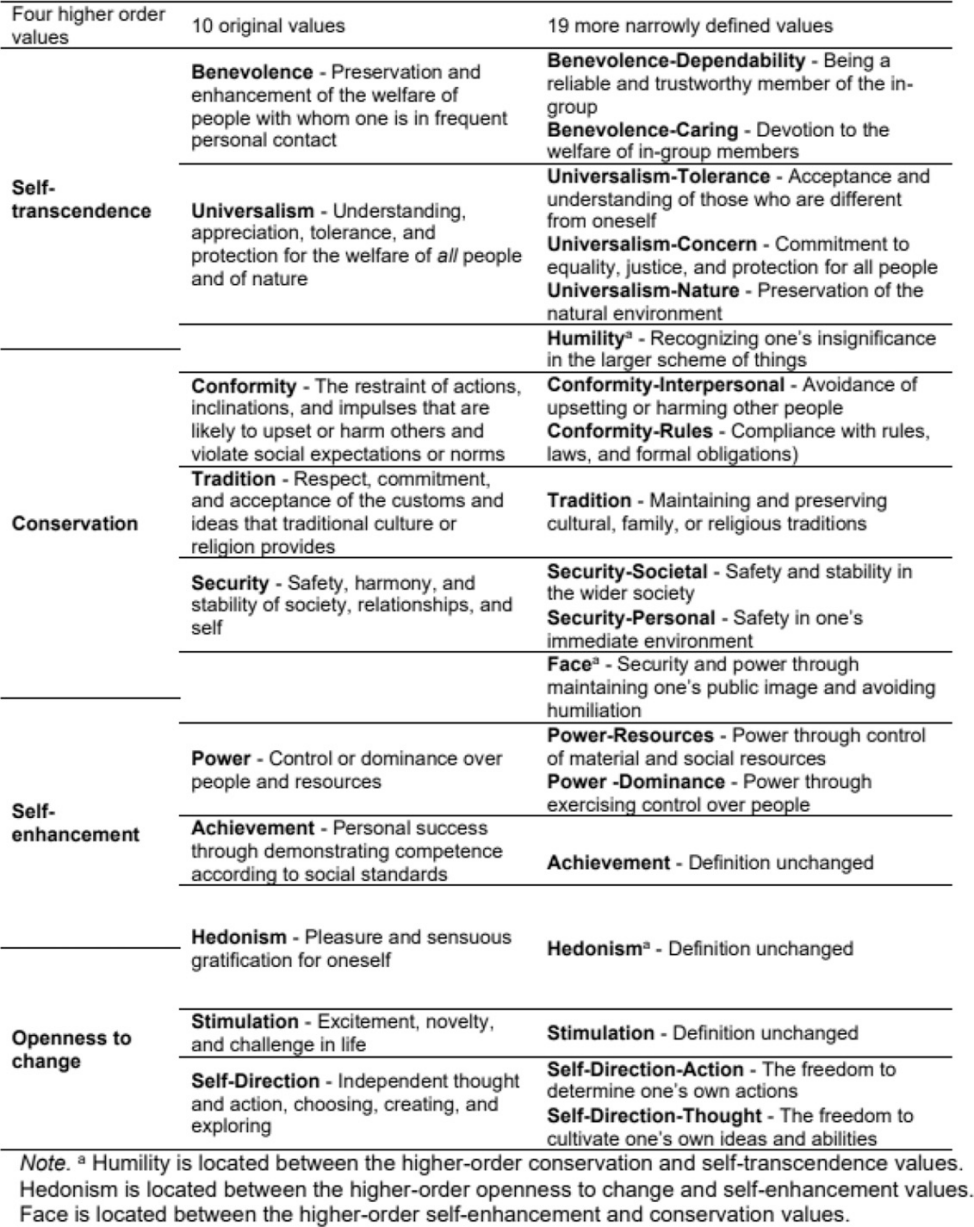

### Iterative adaptation, testing and validation of the conversation instrument

The research team conducted multiple activities to adapt Schwartz’s framework into a conversation instrument suitable for a diverse range nursing home stakeholders.

(1) Translation and language and format adaptation
(2) Expert consultation
(3) ‘Member checking’.

### Translation, language and format adaptation

The research team aimed to develop a conversation instrument with a suitable language level and language use for the stakeholders. To accomplish this, they held several sparring rounds to discuss the English-to-Dutch translation and language level of the values. Additionally, the research team aimed to develop a clear and inviting format for the conversation instrument to facilitate stakeholders’ ability to express their perspectives and the values most important to them. Moreover, we wanted to avoid the need for an extensive explanation of the values, as this could have a negative impact on the quality of conversations. Options for suitable formats were discussed and considered in sparring rounds with the research team.

Comprehensibility and accessibility are necessary conditions to communicate with stakeholders across society.^51^ Therefore, an instrument should use language that is understandable for a diverse range of stakeholders. The B1 language level is assumed to be understandable to a majority of the population.^52,53^ Thus, the formulation of the values and the explanatory sentences were adapted to a B1 language level.

The reports of the research team’s meetings were drawn up by the first author and stored in the research logbook.

### Expert consultation

After this phase, we consulted experts from the Chair of Moral Design Strategy of Fontys University of Applied Sciences. This Chair represents a centre of expertise on the moral challenges associated with the use of technology based on Schwartz’s theory. Its input was crucial as technology may embody features that were neither expected nor foreseen during its development.^31^ Additionally, the expertise of Marieke van Vliet (MvV) one of the authors, was of added value in determining whether the research team’s translation and formulation of the values matched Schwartz’s original meaning, as she is conducting linguistic research on Schwartz’s values. The expert consultation reports were drawn up by the first author and stored in the research logbook.

The first author compared the experts’ feedback with the then current version of the conversation instrument. Subsequently, the expert feedback, together with the current version of the conversation instrument was shared with the research team. The formulation of each value was reviewed and the expert feedback was discussed. The research team jointly decided what adjustments to make to the conversation instrument.

### ‘Member check’

‘Member checking’ is also known as participant or respondent validation^54^ and contributes to the quality of qualitative research.^55^ Our member check it is not used in its original meaning.^56^ Instead, we used the ‘member check’ as content validation to determine whether the value cards are a useful tool in terms of format and content during their development. The ‘member check’ was performed in semi-structured interviews with a diverse range of nursing home stakeholders to determine whether the conversation instrument’s format and language were suitable. The conversation instrument was presented to the participants. From a practice-based case involving the application of surveillance technology they were asked to choose three to four values that were most important to them. Subsequently, they were asked why they had chosen these particular values and whether they perceived that they were sufficiently heard in the values they considered most important. Additionally, participants were asked for feedback on exploring their values with this instrument. This included questions such as follows: ‘Is there anything that you would like to see phrased differently in the conversation instrument’? ‘What suggestions do you have for improving or clarifying the wording’? ‘Is there anything missing in this conversation instrument that you think is important’?

Participants’ feedback on the formulation of these values and the first author’s suggested revisions arising from this feedback were noted in the research logbook and discussed with the research team. The research team jointly decided whether the feedback justified adjusting the formulation for clarity while still adhering to Schwartz’s definitions.

We endorse the inclusion of people with dementia in research, yet cognitive impairments such as reduced comprehension, information processing, and concentration can pose challenges. Based on test conversations and the first author’s experience, the intended use of the value cards proved too demanding for residents with moderate to severe dementia. Instead, simplified questions, derived from the vale cards, were used in accessible conversations with four residents to ensure their perspectives were included. Due to these adaptations, no ‘member check’ was conducted in interviews with residents. However, the perspectives and values of stakeholders including residents will be presented in a subsequent article.

### Participants and stakeholder groups

Participants were selected from three psychogeriatric wards in two healthcare organisations that collaborate with the Academic Collaborative Centre of Older Adults at Tilburg University. The first author contacted the managers of the participating wards, who helped identify relevant stakeholder groups. Snowball sampling was employed to recruit additional participants, ensuring a diverse range of views from individuals directly involved in the application of surveillance technologies.

A total of 34 participants were interviewed, representing various stakeholder groups. The interviews were conducted at a location familiar to the participants. Participant information is presented in Table 2.

**Table 2.** Interview participants.

Stakeholder	Frequency
Informal caregiver	4
Nurse assistant	4
Registered nurse	3
Registered nurse/digicoach	1
Care manager	3
Coordinating practitioner	3
Psychologist	2
Facility staff	5
ICT-employees and application manager	3
Surveillance technology supplier	1
Project leader	1
Occupational therapist	1
Policy officer	1
Board director	2
	Total 34

### Data analysis

With participants’ permission, interviews were recorded with Microsoft Teams with transcription and a separate audio recorder to achieve accurate transcription. The first author compared the Microsoft Teams transcription with the audio-recording, correcting or supplementing it where necessary. By conducting and transcribing the interviews herself, the first author became familiar with the data. Data were analysed using thematic analysis^57^ via Atlas Ti version 24. We coded inductive and axially to integrate codes around central categories and to see which categories were important, how they differed or were related to each other.^58^ The first interview was reviewed by the first and third author to identify codes. Then, the second to twelfth interview were reviewed with the first and second author to continue identifying codes, pursuing consensus on them. If there was any doubt about the codes or quotes in the remaining interviews, the second, third and/ or fifth author were consulted. ‘Member checks’ of the conversation instrument were conducted during the interviews and extracted from the thematic analysis. Data on participants’ reflections on the value cards, some of which also emerged after the interview recording stopped, were noted separately in Word if they were not included in the transcript. All participants’ feedback during the ‘member check’ was presented to the research team and substantively discussed with them in order to jointly consider whether adjustments were justified.

### Ethical considerations

The Ethical Research Board (ERB) of Tilburg University approved this study (ID TSB_RP711). The study design was also presented to the scientific committees of the two participating healthcare organisations, who gave their approval. Participants in the ‘member check’ received detailed study information and gave written informed consent as needed before participating. During the interview, participants were given the option to withdraw from participating without providing a reason. Data were safely stored, and only the research team had access.

## Results

**Figure 3.**
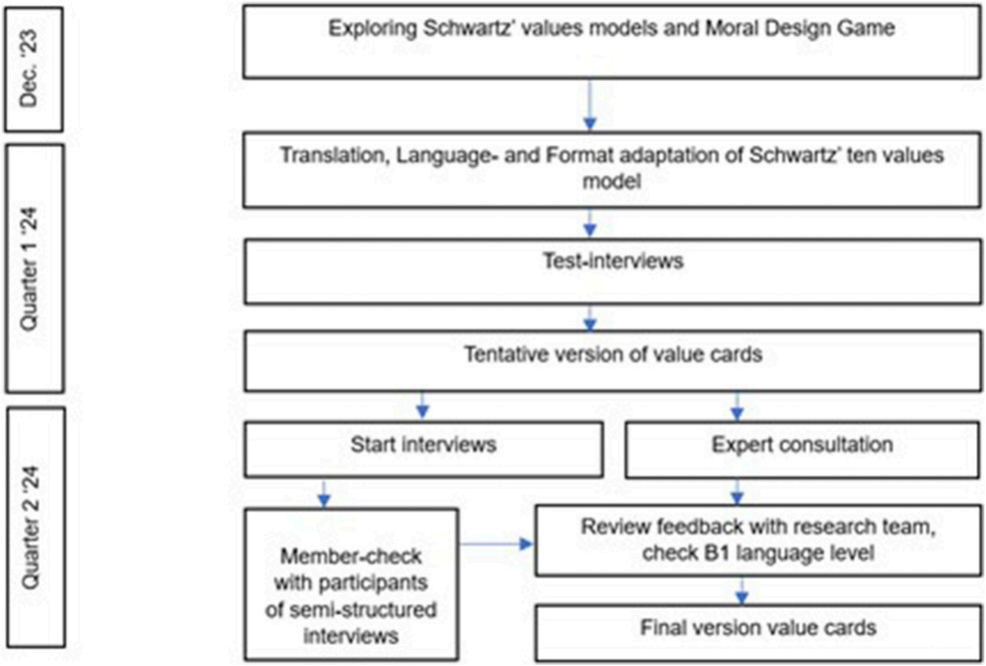
The development process of the value cards.

The results of developing, testing and validating the conversation instrument are presented below in three sub-sections that describe (1) the actions taken when adapting the format and language and the preliminary results of this process, (2) the outcomes of the expert consultations to ensure a proper translation that matched the original meaning of Schwartz’s values and (3) the outcomes of the ‘member checks’. Whenever appropriate, we will refer to citations from the interviews to illustrate the process of member checking. Figure 3 summarises the development of the conversation instrument, which comprises ten individual value cards describing the ten values of Schwartz’s values model tailored to the language level and background of nursing home stakeholders.

### Translation and language and format adaptation

First, we attempted to adjust the circular representation used in Schwartz’s values model^41^ and the Moral Design Game^49^ which derived from Schwartz’s values, and present it with some short explanatory sentences in the circular format in test interviews. However, in sparring sessions with the research team we concluded that this compromised the circular format’s clarity. Additionally, we noted that the circular representation did not give participants sufficient opportunity to absorb the values individually as they were presented all at once. Conversations became too directed and yielded less substantive exploration of perspectives and values, while the intention was to have exploratory in-depth conversations. Insights from test interviews showed improvement when values ​​were presented individually to the participants, with each value explained in a few sentences. To thoroughly explore values and extract the intended insights from interviews, the research team decided to map the values onto ‘cards’ with a few explanatory sentences for each value. These cards could be presented one at a time, allowing the interviewee to absorb the values one by one. This yielded positive results during the test interviews. At the same time, the formulation of each value was reviewed by the research team, in terms of proper translation, language use and B1 language level^52,53^; stakeholder diversity was taken into account. The values and explanatory sentences were formulated in order that, according to the research team, they did justice to the original meaning of Schwartz’s values while simultaneously encouraging participants to reflect on the values most crucial to them. In the sparring sessions with the research team, we discussed and assessed what was proven best in test conversations and jointly determined how to proceed. This iterative process allowed us to respond to what was needed in each phase.

The process of initial translation, determining language use and level according to language level B1 as well as adjusting the format based on the test interviews led to a tentative version of the value cards in March 2024.

It required carefully considered adjustments in format and language, necessitating continuous coordination within the research team. However, an additional check to ensure the translation still aligned with Schwartz’s original intention was not yet performed. Therefore, expert consultation was required.

### Expert consultation

On referral from experts from the Chair of Moral Design Strategy at Fontys University of Applied Sciences, we consulted their colleague, MvV, in April 2024. This latter expert recommended bringing together Schwartz’s ten values model,^41^ the redefined nineteen values model,^39^ the Personal Value Dictionary,^40^ the Portrait Values Questionnaire (PVQ) and Schwartz’s Value Survey (SVS),^50^ comparing the translations and language use prepared by the research team. This comparison led to recommendations for the formulation and explanatory sentences of seven values.

Key considerations of expert feedback included getting to the point more quickly about the meaning of each value according to Schwartz. For example, the value of self-direction is the intrinsic need for autonomy, a more general formulation could trigger the value universalism. For the value of power, feedback was to verify whether the sentence also captures the meaning that it applies to the interviewee themselves. The formulation of the value of conformity should reflect its intention: sacrificing one’s own needs and meeting the expectations of others. For benevolence, it is important that the formulation focus on the internal motivation for doing so, caring, thus distinguishing itself from universalism, which is more focused on its inclusiveness.

The proposed alterations of the formulation and explanatory sentences of the seven values, ‘self-direction’, ‘stimulation’, ‘power’, ‘hedonism’, ‘conformity’, ‘tradition’ and ‘benevolence’, were noted in the research logbook and discussed with the research team. Subsequently, the following adjustments were made:

- The explanatory sentences for the value ‘self-direction’ were adjusted to use first-person pronouns to ensure this value concerned a person’s intrinsic interest.
- The formulation of the value ‘stimulation’ was adjusted to make a clearer distinction between the values ‘stimulation’ and ‘hedonism’.
- The explanatory sentences for the value ‘hedonism’ were altered to be more focused on the respondents themselves to ensure a distinction with ‘benevolence’.
- One explanatory sentence was added and one was adjusted for the value ‘power’ to ensure the sentences addressed what a person finds important in terms of recognition of their person.
- An explanatory sentence for the value ‘conformity’ was reframed to focus more on sacrificing one’s own needs based on obedience/docility.
- Finally, two explanatory sentences for the value ‘benevolence’ were slightly adjusted so that this value focused on a person’s internal motivation to be there for someone else.

In addition, the language level was rechecked to ensure it complied with the intended B1 language level.

### ‘Member check’

The ‘member check’ was performed for each of the 34 interviews. Feedback was provided on the formulation and content for the values ‘security’, ‘self-direction’, ‘stimulation’, ‘hedonism’ and ‘achievement’.

For the value ‘self-direction’, a registered nurse mentioned: *‘The sentence “thinking for yourself and doing it yourself” seems a bit selfish. I’m not like that; it’s not all about me’*.

For the value ‘security’, three participants had feedback, of them, a registered nurse mentioned: *‘Safety is not only about prevention, but also about being able to identify risks in a timely manner’*. Another added: *‘“Using care technology to prevent falls or injuries in residents with dementia”—“preventing” is not possible with care technology, so I would actually like to get rid of that word “prevent”’*.

For the value ‘power’, an employee of the nursing home’s supplier said: *‘The text is a bit long, but the sentence is about being able to participate in decisions about loved ones; that triggers me. The family wants to use a lot of technology, while it is not always necessary […] then you create a false sense of security. The chance of someone falling remains the same’*.

For the value ‘hedonism’, a project leader said: *‘“Enjoying life” […] I don’t know if I would choose the word “enjoy.” […] I haven’t found a word yet that covers it; you don’t need to be entertained every day. Sometimes I just have a bad day’*.

For the value ‘stimulation’, a board director said: ‘*“New things make life more beautiful”—I don’t know if that is the case […] I understand that it is nice to be inspired, but it does not always have to make [life] more beautiful*’.

**Figure 4.**
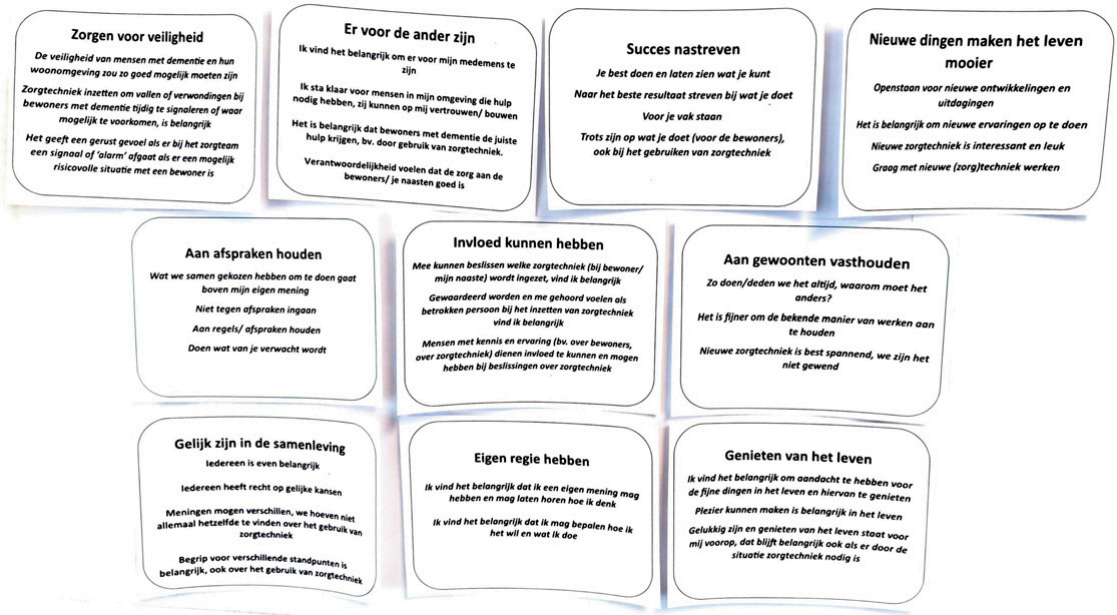
Presentation of value cards in Dutch language level B1.

Participant feedback was discussed during sparring rounds with the research team. Changes were immediately incorporated into subsequent interviews. The title of the value of self-direction was adjusted as there was a consensus that a value should not be formulated in such a way that it triggers a negative meaning such as selfishness as this may affect the interpretation of this value. The formulation of the sentences for the value of security were adjusted to ensure the formulation became more focused on signalling and less on preventing fall incidents, one sentence was removed because it triggered the same unintended meaning. The formulation of other values that received participants’ feedback in the ‘member check’ was discussed. However, no further adjustments were made, for reasons that other formulations had the same meaning, and therefore, adjustments had no added value (value of hedonism); the feedback involved reflected a personal opinion about a value, which is good input, however, does not need to lead to adjustments in the formulation (value of stimulation). Again, the adjustments were checked to ensure the text remained at the B1 language level. This latter version of the value cards was used in the remaining interviews. Adjustments resulted from the first nine interviews, and no further feedback was received from participants after the ninth interview. The feedback on the value cards was mainly positive; the language used on the cards seemed clear to the participants. Table 3 presents an overview of Schwartz’s ten values, the formulation of values and the explanatory sentences. Figure 4 presents the value cards in Dutch with language level B1.

**Table 3.** Adaption to language level B1 of Schwartz’s ten values for conversation instrument.

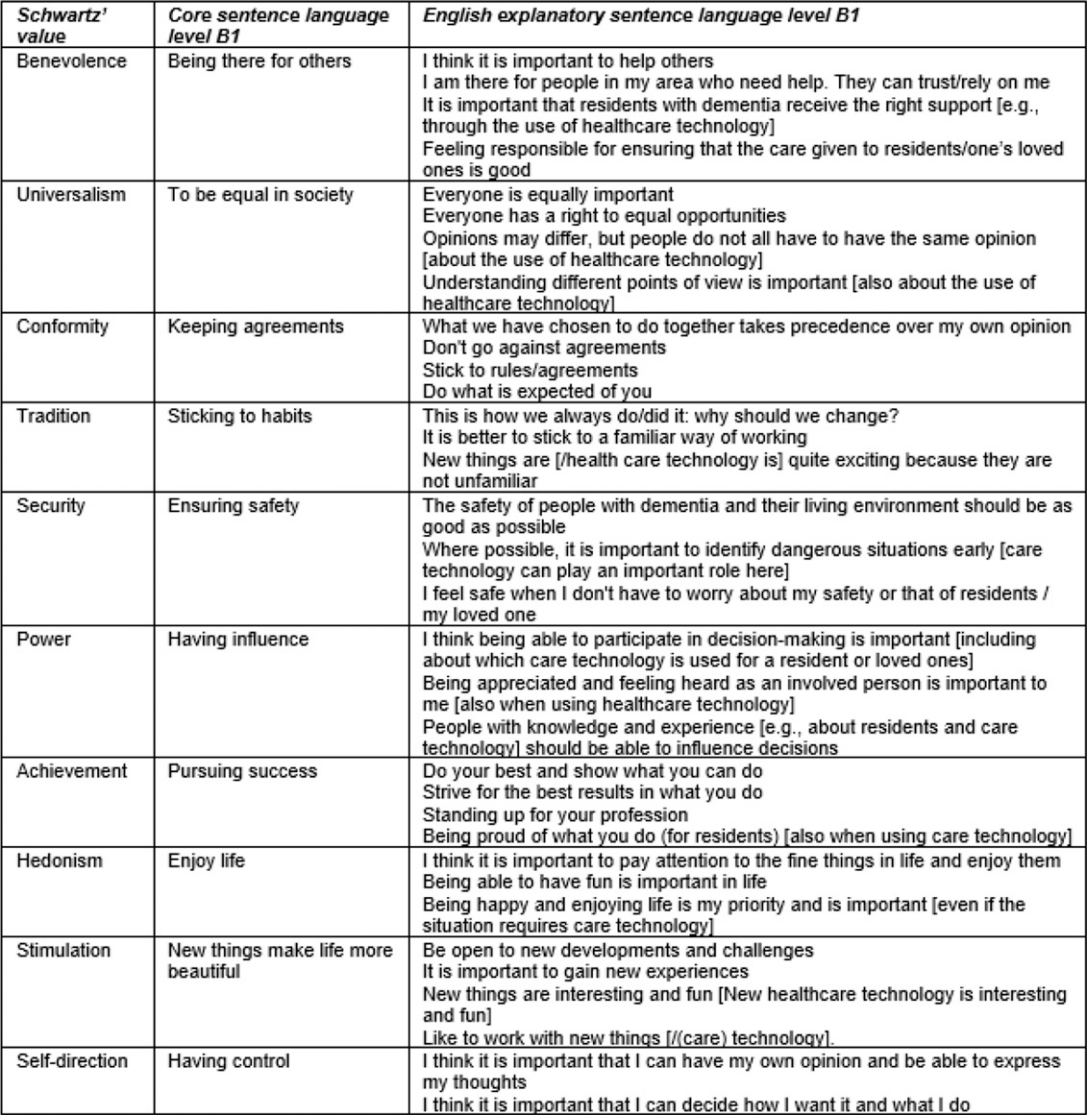

## Discussion

This study aimed to develop a conversation instrument based on Schwartz’s values model to explore values and tailor it to the backgrounds, education levels and language levels of the diverse range of stakeholders involved in the application of surveillance technology in nursing homes.

### Key findings

Our conversation instrument, with which we aimed to conduct in-depth interviews with a broad diversity of stakeholders in a nursing home setting, emerged from an iterative adaptation process based on Schwartz’s basic human values.^39,41^ This process led to ten value cards. This study focused on developing and validating the conversation instrument. The interviews, as well as participants’ feedback, confirmed that using the value cards as conversation instruments enhanced in-depth conversations with representatives of various stakeholder groups. The value cards were helpful in participants to reflect on their perspective, the extent to which they felt involved and revealing what, to them, is most relevant when applying surveillance technologies. This supported them to become aware of underlying values and revealed stakeholder issues that are not easily addressed in non-value-based conversations. Several participants expressed they were enthusiastic about exploring their perspectives with the value cards.

### Relation to existing theories and models

Stakeholder involvement is critical when applying care technologies such as surveillance technologies.^8,28,34,59–61^ Integrating the multiple perspectives of stakeholders contributes to fully understanding the complexities in the application of dementia care technology^8^ and influences the adoption and utilisation of care technologies.^8,62^ In recent years, various implementation theories and tools have emphasised the importance of understanding stakeholders’ perspectives and values and the relevance of their involvement and commitment. This is reflected in theories such as the Triple I model,^60,63^ the Non-adoption, Abandonment, Spread, Scale-up and Sustainability (NASSS) framework^28,64^ and the Normalisation Process Theory (NPT).^59,65^ These theories, models or frameworks undeniably contribute to the sustainable implementation of care technologies. While these models contribute to understanding the relevance of stakeholder involvement in implementation processes, they do not offer tools for directly exploring individual stakeholder values.

### Current position of values in health care

The word ‘value’ has different meanings. For example, the value proposition, as referred to in the NASSS framework, is a statement of the value that a technology might generate for suppliers or users.^28^ In the context of the valuation of technologies by stakeholders, the costs of care technologies versus benefits and returns are mapped in the Vilans’ Value Model.^66^ This model highlights ten themes from the perspective of different stakeholders and is recommended for measuring stakeholders’ values.^66^ Another model, Value-Sensitive Design (VSD), aims to give designers an understanding of users’ values, needs and practices.^67,68^ The VSD toolkit was developed for use in the design phase and features envision cards that stimulate creative exploration and evoke discussions about users’ concerns in the context of design and engineering.^67,69^ Similarly, Shared Decision-Making (SDM) creates insight into a person’s most relevant values and motives and is used to facilitate in-depth conversations.^70,71^ An example of SDM is a joint decision-making process between a care provider (or another professional) and a patient.^71^ SDM explores perspectives and values of patients or care recipients, and supports individuals in making a decision, usually in a medical setting. SDM does not specifically include people with cognitive problems such as dementia, nor does it focus on a multi-stakeholder approach.

Overall, while these models emphasise the importance of understanding stakeholder values, they are often limited to the design phases of technology, such as with VSD. Moreover, none focuses specifically on understanding stakeholders’ values when implementing newly developed or existing technology in care practice, nor are any designed for conversations with the multiple stakeholders in (psychogeriatric) nursing homes. In addition, while the Vilans’ Value Model recommend measuring stakeholders’ values,^66^ they do not provide a practical tool for this. Although increasing value is attached to stakeholder values, there is still a lack of practical instruments for in-depth conversations about these values in care practice. If we want to focus on the involvement of multiple stakeholders in the pursuit of high-quality, person-centred care, it is relevant to know the perspectives of stakeholders and to recognise their values. A tool to explore stakeholder values is therefore relevant. Our study addresses this gap by providing a practical tool, value cards, for exploring values in multi-stakeholder contexts. These value cards go beyond existing frameworks by enabling in-depth conversations about (individual) stakeholders’ intrinsic motivations and concerns, making them a suitable tool for exploring stakeholders’ values.

## Practical implications

The reasons why collaboration and communication between various stakeholders is sometimes challenging are not always obvious, nor are they always clear to those involved themselves.^61^ Stakeholders’ perspectives on ethical issues, such as the use of surveillance technologies, are influenced by their values, which are often multifaceted and vary per stakeholder involved.^66^ People often do not explicitly express the values that are important to them; however, these will influence their behaviour and attitude.^44,60,72^ When value conflicts emerge between organisational technology decisions and stakeholder values, our findings suggest several approaches. First, the value cards help identify specific conflicts early, before positions become fixed. For example, if families interpret ‘security’ as continuous monitoring while care professionals focuses on maintaining dignity, the cards reveal different understandings. Second, the tool facilitates exploring common ground. Stakeholders often share underlying values,^61^ which provided a foundation to move forward together. Rather than expecting alignment with employer values, sustainable technology adoption demands value exploration and acknowledgement across stakeholder groups. Organisations could adapt implementation strategies when differences in values lead to concerns about the application of surveillance technology. This process involves multi-stakeholder discussions acknowledging organisational goals, employee concerns, residents’ preferences, and family members’ expectations. In turn, this can lead to approaches that respect shared values as well as differences in values among stakeholders while simultaneously meeting diverse needs. As Wouters (2024) mentioned: the shared values ​​between various stakeholders provide an opening for connection, and this connection has major added value when applying care technologies.

This can then lead to approaches that respect shared values as well as differences in values, while meeting diverse needs.

## Broader implications

The value-based conversation approach may offer insights for ethical technology assessment beyond surveillance technologies, as it provides a structured way to explore stakeholder values in ethical dilemmas in the implementation of technological innovations. Considerations about the application of surveillance technologies and stakeholder involvement affect more than just the implementation process. These technologies will continue to be applied for residents, affecting new stakeholders who have not gone through the implementation process themselves. Each of these new stakeholders will have to be included in this process so that they, too, feel heard.

Several business studies emphasise the need for multiple-stakeholder collaboration.^73^ However, multi-stakeholder collaborative processes in the business field are known to be extremely difficult in practice as stakeholders frequently have different priorities and value logics. Additionally, business companies find boundary crossing challenging, which involves learning from and co-creating with others outside one’s own work field or personal situation.^74^ The relevance of boundary crossing is also mentioned by Bühler^75^ who found that dementia care stakeholders emphasised the importance of adapting their language to various target audiences and their needs. Continuous and transparent communication is recommended to manage mutual expectations and contribute to stakeholders’ commitment.^75^ Contemporary technological innovations can only succeed through close interrelationships within the social domain.^61,62^ This challenges people to look outside their own work fields or personal situations to include other perspectives, which is a dire necessity.^62^

### Strengths and limitations

Several steps were taken throughout this study to ensure the quality and relevance of the value cards. One of the main strengths was that the research team consulted an expert to ensure that the content of the value cards matched the original meaning of Schwartz’s values. In addition, we used a ‘member check’ with interview participants to ensure that the formulation was clear and understandable for a diverse range of stakeholders.

Another strength was the focus on accessibility. The translation at the B1 language level prevented the use of language that was too complex for a diverse population. According to the Common European Framework of Reverence (CEFR), B1 is the language level of people who are able to read texts with concrete topics in a foreign language, without the need for visual support.^51^ It is equivalent to International Adult Literacy Survey (IALS) level 2,^51^ which 90% of the Dutch population has mastered.^76^ The IALS is specifically aimed at the language level of adults in their native language.^51^ For this article, which is aimed at an international audience, we made an additional English translation of our value cards. A proofreading certificate is available to certify that the English-language version is at a B1 language level. However, despite these efforts, it is conceivable that further testing and validation of our value cards’ format and formulation will be needed in future research, this may also be in different contexts and for different topics.

The value cards in the interviews were very promising in exploring stakeholder values and offer participants an instrument for reflection on values. However, this study solely focused on the development of the value cards in a participatory design with experts and stakeholders from nursing home practice. The development and testing in one setting represents a limitation. In our opinion, the value cards or a tool based on that, could be helpful for use with other heterogeneous stakeholder groups to facilitate in-depth conversations on dilemmas or delicate issues, to raise awareness of stakeholders’ values. This can be a first step towards shared decision-making. In addition, it could be a topic of further research. These value cards could also be applied in a multi-stakeholder setting on topics other than the use of surveillance technologies. We think it is worthwhile to explore this in further research. A point to note here is that using the value cards in practice demands knowledge of Schwartz’ theory of human values from the discussion leader and calls upon the communication skills and time of the discussion leader.

Although Schwartz’s value model is a validated instrument for measuring basic human values, and many studies have used questionaires such as PVQ (Portrait Values Questionnaire) and SVS (Schwartz’s Value Survey) to assess values in psychology, sociology and economics, little qualitative research has been conducted exploring values in interviews without using closed-ended Likert-scale questionnaires. In our opinion, it is worthwhile to further explore the application of Schwartz’ values model in qualitative interviews, encouraging in-depth conversations about values to capture different perspectives in multi-stakeholder settings and achieve insights that transcend perspectives and professions. Specifically this could provide a starting point for in-depth conversations that focus on different perspectives and sheds light on reasons for tensions that differences in values could evoke.

Finally, the development of the value cards took considerably longer than anticipated. We expected we could easily adapt the values model for use in interviews with a broad diversity of stakeholders. However, it required carefully considered adjustments in format and language, necessitating continuous coordination within the research team, the experts, and ‘member checking’ during the interviews.

## Conclusion

This article describes the iterative development process of a conversation instrument derived from Schwartz’s basic human values for exploring the values of the various stakeholders involved in the application of surveillance technologies for people with dementia in nursing homes. The conversation instrument, which consisted of ten value cards, was used in in-depth conversations and was proven to help stakeholders share their perspectives and values about the application of surveillance technologies for people with dementia in nursing homes. Additionally, the value cards inspired stakeholder reflection on what they considered the most important factors in applying surveillance technology. This knowledge, along with information about the participants’ shared values, provides an opening for interconnection between multiple stakeholders, which is of added value when applying care technologies.

## Supplemental Material

Supplemental Material - Facilitating value-driven conversations: Development of a conversation instrument for surveillance technology use in nursing homes Supplemental Material for Facilitating value-driven conversations: Development of a conversation instrument for surveillance technology use in nursing homes by Daniëlle van Gaans-Riteco, Annerieke Stoop, Irene Muller-Schoof, Marieke van Vliet, Eveline Wouters in Nursing Ethics.

## Data Availability

The dataset is not publicly available due to the sensitive nature of the data. All data is stored in the digital project file ‘Domotica Psychogeriatrie 4200P173’ of Tranzo, Tilburg University, in accordance with the Data Handling and Methods Reporting.*
