# Supplementary material for: An instrument to facilitate value-driven conversations on surveillance technology
Source: Nurs Ethics. 2025 Sep 25;32(8):2530–50. doi: 10.1177/09697330251376894 (PMC12644261; doi:10.1177/09697330251376894)
Supplement: Supplemental Material - Facilitating value-driven conversations: Development of a conversation instrument for surveillance technology use in nursing homes [file sj-pdf-1-nej-10.1177_09697330251376894.pdf]

Theoretical sources and their content for adaptation process of value cards *

*For sequence adaptation process read table from left to right

Blue= social focus	yellow= personal focus

Category	Motivational Type	Value 10 value model (Schwartz, 2012a)	Value 19 value model (Schwartz, 2012b)	Definition (Schwartz, 2012a, 2012b)	Explanation (Schwartz, 2012a, 2012b)	Personal Values Dictionary (Ponizovskiy, 2020)	SVS (Schwartz Value Survey) (Schwartz, 2012b)	PVQ (Portrait Values Questionnaire) (Schwartz, 2021)	English to Dutch Translation of PVQ (Schwartz, 2021)	Values of Schwartz in Dutch applicable for interviews (language level B1)	Values of Schwartz in English applicable for interviews (language level B1)
Growth-self expansion Anxiety free	Openness to change	Self-direction	Self-direction-thought	Freedom to cultivate one's own ideas and abilities	Creativity, imagination, curious, interested, being independent	ability, act, acted, acting, action, actions, activist, activities, activity, acts, aim, analysis, artistic, awareness, backbone, brilliance, brooding, choice, choose, claiming, clever, college, confident, confrontation, contemplation, controversial, controversy, conviction, create, creates, creation, creative, creativity, critically, curious, dash, decision, defiance, determination, determined, develop, devotion, direct, directing, direction, discovery, dispute, disregard, distinctive, education, educational, effort, efforts, enlightenment, evolving, famous, free, freedom, freely, goal, goals, grit, headway, hobby, hunch, idea, ignorance, imagination, inclination, independence, independent, individual, individuality, ingenuity, initiative, insight, inspiration, inspired, inspiring, instinct, intellect, intellectual, intelligence, intelligent, intend, intends, intention, interest, intuition, knowing, knowledge, learn, learned, learning, lessons, logic, mature, meditation, mind, minded, observation, obsessed, obsession, opinion, original, philosophy, plan, pluck, pondering, principles, protest, purpose, rationale, reasoning, reflection, resolve, revolt, scholar, schooling, science, scrutiny, seriousness, sceptical, smart, special, speculation, spirit, talent, talents, think, thinking, thinks, thought, thoughtful, thoughts, undertaking, vanity, vision, willpower, wit	Creativity, imagination, curious, interested	It is important to him:<br>-to form his views independently<br>-to develop his own opinions<br>-to figure out things for himself	Het is belangrijk voor hem:<br>-om onafhankelijk zij eigen beeld te vormen<br>-om zijn eigen mening te ontwikkelen<br>-om dingen zelf uit te zoeken	*Eigen regie*<br>Ik vind het belangrijk dat ik een eigen mening mag hebben en mag laten horen hoe ik denk<br><br>Ik vind het belangrijk dat ik mag bepalen hoe ik het wil en wat ik doe	*Having control (Self-direction)*<br>(Language level B1 for: I have the freedom to cultivate my own ideas and determine my actions)<br><br>I think it is important that I can have my own opinion and be able to express my thoughts<br><br>I think it is important that I can decide how I want it and what I do
			Self-direction Action	Freedom to determine one's own actions	Choosing your own goals, own purposes, being independent, self-reliant		Choosing your own goals, own purposes, independent/ self-reliant	It is important to him:<br>-to make his own decisions about his life<br>-to plan his activities independently<br>-to be free to choose what he does by himself	Het is belangrijk voor hem:<br>-om zijn eigen beslissingen te maken<br>-om onafhankelijk zijn eigen activiteiten te plannen<br>-om de vrijheid te hebben om zelf te kunnen kiezen wat hij wil doen		
Growth-self expansion Anxiety free		Stimulation	Stimulation	Excitement, novelty and change	Excitement with stimulating experiences, a varied life with novelties, changes and challenges doing different things, seeking adventures	adrenaline, adventure, adventures, amusement, amusing, attempt, attempted, attempting, attempts, audacity, bored, boredom, brave, breakthrough, breathtaking, bustle, challenge, challenged, challenges, challenging, changes, contradiction, courage, coward, curiosity, dare, daring, different, discover, discovered, discoveries, discovering, disturbance, drama, dramatic, dramatically, drive, dynamic, dynamics, eagerness, endeavour, energetic, energies, energy, enthusiasm, enthusiastic, excitement, exciting, exercise, experiences, experiment, exploration, explorer, exploring, extraordinary, extreme, fascinating, fervour, flurry, frenzy, fuss, gamble, glamour, hustle, hysteria, impatience, impulse, innovative, instability, intensity, intensive, interesting, intriguing, invention, inventions, irregular, motivation, movement, mysterious, newer, newest, newly, novel, novelty, opportunities, opportunity, paranoid, passion, passionate, progressive, radical, revolutionary, risk, risks, shock,	An exciting life/ stimulating experiences, a vierd life/ novelty and change, daring/ seeking adventure	It is important to him:<br>-always look for different things to do<br>-to take risks that make life exciting<br>-to have all sorts of new experiences	Het is belangrijk voor hem:<br>-om altijd naar andere dingen te kijken om te doen<br>-om risico's te nemen die zijn leven opwindend maken<br>-om allerlei nieuwe ervaringen op te doen	*Nieuwe dingen maken het leven mooier*<br>Openstaan voor nieuwe ontwikkelingen en uitdagingen<br><br>Het is belangrijk om nieuwe ervaringen op te doen<br><br>Nieuwe zorgtechniek is interessant en leuk<br><br>Graag met nieuwe (zorg)techniek werken	*New things make life beautiful*<br>Be open to new developments and challenges<br><br>It is important to gain new experiences<br><br>New things are interesting and fun [New healthcare technology is interesting and fun]<br><br>Like to work with new things [/(care) technology].

						shocked, spontaneous, stimulated, stimulating, stir, strain, striving, struggle, surprising, surprisingly, temper, temperament, tension, thrill, turmoil, uncertain, uncertainty, unfamiliar, unique, unprecedented, unpredictable, unstable, variety, venture, vitality					
	**Motivational Type**	**Value 10 value model (Schwartz, 2012a)**	**Value 19 value model (Schwartz, 2012b)**	**Definition (Schwartz, 2012a, 2012b)**	**Explanation (Schwartz, 2012a, 2012b)**	**Personal Values Dictionary (Ponizovskiy, 2020)**	**SVS (Schwartz Value Survey) (Schwartz, 2012b)**	**PVQ (Portrait Values Questionnaire) (Schwartz, 2021)**	**English to Dutch Translation of PVQ (Schwartz, 2021)**	**Values of Schwartz in Dutch applicable for interviews (language level B1)**	**Values of Schwartz in English applicable for interviews (language level B1)**
	Self-transcendence	x	Humility	Recognizing one's significance in the larger scheme of things	being humble, modest, accepting your portion in life, submitting to life's circumstances and being satisfied with what one has	x	n.a.	It is important to him:<br>-to never think he deserves more than other people<br>-to be humble<br>-to be satisfied with what he has and not ask for more	Het is belangrijk voor hem:<br>-om nooit te denken dat hij meer waard is dan andere mensen<br>-om bescheiden te zijn<br>-om tevreden te zijn met wat hij heeft en niet om meer te vragen	nvt	
		Benevolence	Benevolence (dependability)	Being a reliable and trustworthy member of an ingroup	Being responsible, loyal and faithful to friends, referring to relations with friends and less to family than caring does	acquaintance, advice, affection, aid, ally, assist, assistance, association, backing, belonging, brotherhood, care, caring, closeness, communal, companion, companions, companionship, compassion, compassionate, concern, confidence, contribution, coop, cousin, delicate, dependable, empathy, encourage, encouraged, encouragement, encouraging, families, family, father, feeling, fidelity, fondness, forgive, forgiveness, forgiving, friend, friendly, friends, friendship, generosity, gentle, genuine, gift, goodness, goodwill, grandma, grandmother, grandparents, guidance, help, helpful, helping, honest, hospitality, husband, intimacy, kindly, kindness, love, loyal, loyalty, mom, mother, need, neighbourhood, neighbours, nurture, parents, participation, pity, relationship, reliable, rely, responsibilities, responsibility, responsible, sincere, sorrow, support, supporting, supportive, sympathetic, sympathy, tenderness, thanked, treatment, trustworthy, warmth, wife	responsible/dependable, loyal/faithful to friends	It is important to him:<br>-that people he knows have full confidence in him<br>-to be a dependable and trustworthy friend	Het is belangrijk voor hem:<br>-dat al zijn vrienden en familie volledig op hem kunnen vertrouwen<br>-om een betrouwbare en geloofwaardige vriend te zijn	*Er voor de ander zijn*<br>Ik vind het belangrijk om er voor mijn medemens te zijn<br><br>Ik sta klaar voor mensen in mijn omgeving die hulp nodig hebben, zij kunnen op mij vertrouwen<br><br>Het is belangrijk dat bewoners met dementie de juiste hulp krijgen, bijvoorbeeld door gebruik van zorgtechniek.<br><br>Verantwoordelijkheid voelen dat de zorg aan de bewoners/ je naasten goed is	*Being there for others*<br>I think it is important to help others<br><br>I am there for people in my area who need help. They can trust/rely on me<br><br>It is important that residents with dementia receive the right support [e.g., through the use of healthcare technology]<br><br>Feeling responsible for ensuring that the care given to residents/one's loved ones is good
			Benevolence (caring)	Devotion to the welfare of ingroup members	Being helpful, care for wellbeing of loved ones, being honest, help near ones. Being empathic, supporting and assisting those in need, feeling responsibility for others		helpful/working for others welfare, honest/genuine, forgiving/willing to pardon	It is important to him:<br>-to take care of people he is close to<br>-to help people dear to him<br>-to concern himself with every need of his dear ones	Het is belangrijk voor hem:<br>-om voor mensen te zorgen die hem dierbaar zijn<br>-om mensen te helpen die dierbaar voor hem zijn<br>-om zich te bekommeren om elke behoefte van zijn dierbaren		
		Universalism	Universalism (concern)	Commitment to equality, justice and protection of all people	Equality (in opportunities) for all, social justice, world peace, protect the weak	accept, acceptance, acknowledge, acknowledgement, acknowledging, address, aesthetic, agreement, amends, assortment, balance, balancing, beautiful, beautifully, beauty, charitable, climate, coalition, collaboration, communion, communities, community, company, complex, compromise, connection, cooperation, cooperative, coordinated, cruel, culture, decorum,	equality for all, social justice, world at peace	It is important to him:<br>-that the weak and vulnerable in society are protected<br>-that every person in the world have equal opportunities	Het is belangrijk voor hem:<br>-dat de zwakken en kwetsbare mensen in de samenleving beschermd worden<br>-dat iedere persoon op de wereld gelijke mogelijkheden heeft	*Gelijk zijn in de samenleving*<br>Iedereen is even belangrijk<br><br>Iedereen heeft recht op gelijke kansen<br><br>Meningen mogen verschillen, we hoeven niet allemaal hetzelfde te vinden over het	*Equality in society*<br>Everyone is equally important<br><br>Everyone has a right to equal opportunities<br><br>Opinions may differ, but people do not all have to

						democracy, democratic, democrats, discrimination, diverse, diversity, empowered, environment, environmental, equality, explanation, fair, fairness, fellowship, fusion, generous, graceful, greed, greedy, humanity, injustice, interpretation, justice, liberal, liberation, liberties, liberty, likeness, mankind, meaning, meaningful, mercy, natural, nature, peace, peaceful, protect, protected, protecting, protection, protects, realization, rights, sacrifice, serenity, share, sharing, sincerity, social, socially, society, solidarity, spiritual, tact, tolerance, tolerant, tolerate, tolerated, understand, understanding, unfair, union, united, unity, universal, vulnerable, welcome, welcoming, wisdom, wise, wisely		lin life<br>-that everyone be treated justly, even people he doesn't know	in het leven<br>-dat iedereen rechtvaardig behandeld wordt, zelfs mensen die hij niet kent	gebruik van zorgtechniek<br><br>Begrip voor verschillende standpunten is belangrijk, ook over het gebruik van zorgtechniek	the same opinion [about the use of healthcare technology]<br><br>Understanding different points of view is important [also about the use of healthcare technology]
			Universalism (Nature)	Preservation of the natural environment	Protect the environment, unity with nature, world beauty, adapt/ fit into nature		protect the environment, unity with nature, world beauty	It is important to him:<br>-to care for nature<br>-to take part in activities to defend nature<br>-to protect the natural environment from destruction of pollution	Het is belangrijk voor hem:<br>-om voor de natuur te zorgen<br>-om deel te nemen in activiteiten om de natuur te beschermen<br>-om de natuurlijke omgeving te beschermen tegen vernieling of vervuiling		
			Universalism (tolerance)	Acceptance and understanding of those who are different from oneself	Being broadminded, tolerant, mature understanding, listen to people who are different, understand those who disagree		broadminded/tolerant, wisdom/mature understanding	It is important to him:<br>-to be tolerant toward all kind of people and groups<br>-to listen and understand people who are different from him<br>-to accept people even when they disagrees with them	Het is belangrijk voor hem:<br>-om tolerant te zijn tegenover allerlei soorten mensen en groepen<br>-om naar mensen die anders zijn dan hem te luisteren en te begrijpen<br>-om mensen te accepteren zelfs als hij het oneens is met hen		
**Categories**	**Motivational Type**	**Value 10 value model (Schwartz, 2012a)**	**Value 19 value model (Schwartz, 2012b)**	**Definition (Schwartz, 2012a, 2012b)**	**Explanation (Schwartz, 2012a, 2012b)**	**Personal Values Dictionary (Ponizovskiy, 2020)**	**SVS (Schwartz Value Survey) (Schwartz, 2012b)**	**PVQ (Portrait Values Questionnaire) (Schwartz, 2021)**	**English to Dutch Translation of PVQ (Schwartz, 2021)**	**Values of Schwartz in Dutch applicable for interviews (language level B1)**	**Values of Schwartz in English applicable for interviews (language level B1)**
Self-protection-anxiety-avoidance	Self-enhancement	Hedonism	Hedonism	Pleasure and sensuous gratification	Derives from organic needs and the pleasure associated with satisfying them (enjoying life, pleasure)	allure, appeal, appetite, attraction, celebrations, charm, cheer, cheerful, comedy, comfort, comfortable, compensation, contentment, courting, craving, delicious, delight, delighted, delightful, delights, desire, diversion, ease, ecstasy, elation, enchantment, enjoy, enjoyed, enjoying, enjoyment, enjoys, entertain, entertaining, entertainment, erotic, euphoria, fascination, fetish, flavor, fulfillment, fun, funny, games, glee, gratification, happier, happily, happiness, happy, hilarious, holiday, humor, indulgence, jest, joke, joking, joy, kidding, laughter, leisure, lightness, longing, lure, lust, mirth, optimism, orgasm, party, play, playful, pleasant, pleasing, pleasure, pleasures, recreation, refreshment, rejoicing, relax, relaxation, relish, rest, satisfaction, satisfying, savor, seduction, sensation, sensual, sex, sexual, sexuality, sexually, sexy, sparkle, temptation, thirst, unpleasant, vacation	n.a.	It is important to him:<br>-to have a good time<br>-to enjoy life's pleasures<br>-to take advantage of every opportunity to have fun	Het is belangrijk voor hem:<br>-om het naar zijn zin te hebben<br>-om van de geneugten van het leven te genieten<br>-om van elke gelegenheid gebruik te maken om plezier te maken	*Genieten van het leven*<br>Ik vind het belangrijk om aandacht te hebben voor de fijne dingen in het leven en hiervan te genieten<br><br>Plezier kunnen maken is belangrijk in het leven<br><br>Gelukkig zijn en genieten van het leven staat voorop, dat blijft belangrijk ook als er door de situatie zorgtechniek nodig is	*Enjoy life*<br>I think it is important to pay attention to the fine things in life and enjoy them<br><br>Being able to have fun is important in life<br><br>Being happy and enjoying life is my priority and is important [even if the situation requires care technology]
		Achievement	Achievement	Success	Pursuing success	accomplish, accomplished, achieve,	Successful/	It is important to	Het is belangrijk voor	*Succes nastreven*	*Pursuing success*

				according to social standards	as judged by the normative standards of one's culture<br>*Personal success<br>*Demonstrating competence and performance in concrete action, obtaining social approval	achievement, achieving, advance, advancement, advantage, appreciate, appreciation, approval, attained, best, biggest, brains, business, capabilities, capability, capable, capacity, celebrate, champion, compete, competence, competent, competing, competition, competitive, completion, craft, defeat, development, dignity, effective, efficiency, efficient, elegance, employment, experience, expertise, facility, greatest, growth, honors, improvement, improvements, improving, industry, job, jobs, loose, losing, mastery, merit, perfection, perform, popular, pride, productive, professional, progress, promoted, promotion, proud, qualified, quality, readiness, recognize, recognized, recognizes, results, reward, rewards, skill, skills, succeed, succeeded, succeeding, success, successes, successfully, top, training, win, won, work, worth, yield	achieving goals, ambitious/ aspiring, capable/ competent. No clearly indication whether success is judges internally or externally. We narrow the original definition of achievement is express the underlying motivation to be judged as successful by others (dropping the concept of competence)	him:<br>-to have ambitions in life<br>-to be very successful<br>-that people recognize what he achieves	hem:<br>-om ambities te hebben in het leven<br>-om zeer succesvol te zijn<br>-dat mensen erkennen wat hij bereikt	Trots zijn op wat je doet (voor de bewoners), ook bij het gebruiken van zorgtechniek<br><br>Je best doen en laten zien wat je kunt<br><br>Naar het beste resultaat streven bij wat je doet<br><br>Voor je vak staan	Do your best and show what you can do<br><br>Strive for the best results in what you do<br><br>Standing up for your profession<br><br>Being proud of what you do (for residents) [also when using care technology]
Self-protection-anxiety-avoidance		Power	Power (Dominance)	Power via control over people	Dominance over people, power to constrain others to do what one wants	acquire, agency, aggressive, ahead, attack, authority, award, benefit, bonus, caliber, capital, cash, command, commerce, conquer, conquest, contest, control, controlling, dealing, demand, demands, dominance, dominant, dominate, dominion, earning, earnings, economics, edge, elevation, elite, enforced, enforcement, entitled, expensive, fancy, fight, force, forcing, fortune, gain, gaining, humiliation, income, influence, influential, interests, lead, leadership, luxury, management, manipulation, might, mighty, money, monopoly, ownership, platinum, position, potential, power, powerful, privilege, proceeds, profit, profitable, profits, property, prosperity, purchase, purchases, pursuit, resources, revenue, rich, richest, salary, selfish, spine, standing, status, strength, strengthened, strengths, strong, superior, superiority, supervision, surplus, sway, trade, utility, vantage, victory, wages, weak, weaker, weakness, weaknesses, wealth, wealthy	Social power/control over others, authority/ right to command	It is important to him:<br>-that people do what he says they should<br>-to have the power to make people do what he wants<br>-to be the one who tells others what to do	Het is belangrijk voor hem:<br>-dat mensen doen wat hij ook zegt dat ze zouden moeten doen<br>-om de macht te hebben om mensen te laten doen wat hij wil<br>-om diegene te zijn die zegt wat anderen moeten doen	*Invloed kunnen hebben*<br><br>Mee kunnen beslissen welke zorgtechniek (bij bewoner/ mijn familielid) ingezet wordt, vind ik belangrijk<br><br>Gewaardeerd worden en me gehoord voelen als betrokken persoon bij het inzetten van zorgtechniek vind ik belangrijk<br><br>Mensen met kennis en ervaring (bijvoorbeeld over bewoners met dementie, over zorgtechniek etc.) dienen invloed te kunnen en mogen hebben bij beslissingen over zorgtechniek.	*Having influence*<br>I think being able to participate in decision-making is important [including about which care technology is used for a resident or loved ones]<br><br>Being appreciated and feeling heard as an involved person is important to me [also when using healthcare technology]<br><br>People with knowledge and experience [e.g., about residents and care technology] should be able to influence decisions
			Power (Resources)	Power by control of material and social resources	Power to control events through one's material assets		Wealth/ material possessions	It is important to him:<br>-To have the power that money can bring<br>-to be wealthy<br>-to own expensive things that show his wealth	Het is belangrijk voor hem:<br>-om geld te hebben omdat het macht geeft<br>-om rijk te zijn<br>-om dure dingen te bezitten en rijkdom te laten zien		
		x	Face	Security and power by maintaining one's public image, avoiding humiliation	Maintaining and protecting prestige<br><br>*expresses both value and security values*	X	Social recognition/ respect, preserving public image/ maintaining face	It is important to him:<br>-that no one should ever shame him<br>-to protect his public image<br>-to never be humiliated	Het is belangrijk voor hem:<br>-dat niemand hem ooit openbaar in verlegenheid zou brengen<br>-om zijn publieke imago te beschermen<br>-om nooit vernederd te worden	nvt	
	Conservation	Security	Security (Personal)	Safety in one's immediate environment	The sense of belonging, feeling others care about me, secure surroundings assure safety for loved ones. Taking precautions to prevent harm,	afraid, alarm, alert, armor, attention, barrier, beware, calculation, calm, calmness, camouflage, careful, catastrophic, caution, cautious, clean, consequences, crisis, custody, damages, danger, dangerous, defend, defending, defense, defenses, defensive, disease, enemies, foresight, guarantee, guard, guardian, guarding, harm, harmless, health, heed, hiding, insurance, menace, neat, order, precautions, preserving, prevent, privacy,	Sense of belonging, feeling others care about me, healthy/ not sick, reciprocating favors/ avoiding indebtedness, clean/ neat, tidy	It is important to him:<br>-to avoid disease and protect his health<br>-to be personally safe and secure<br>-to avoid anything dangerous	Het is belangrijk voor hem:<br>-om ziekte te vermijden en zijn gezondheid te onderhouden<br>-om persoonlijk veilig en zeker te zijn<br>-om iets gevaarlijks te vermijden	*Zorgen voor veiligheid*<br>De veiligheid van mensen met dementie en hun woonomgeving zou zo goed mogelijk moeten zijn<br><br>Zorgtechniek inzetten om vallen of verwondingen bij bewoners met dementie tijdig te signaleren of indien	*Ensuring safety*<br>The safety of people with dementia and their living environment should be as good as possible<br><br>Where possible, it is important to identify dangerous situations early [care technology can play

					being warned in case of threats<br>Also expresses benevolence	prudent, quiet, refuge, retreat, safe, safely, safer, safety, salvation, save, scare, scared, secure, secured, securely, securing, security, shelter, shield, silence, stability, stable, stealth, survival, surviving, threat, threaten, threatened, threatening, threats, tidy, tranquility, trouble, violence, ward, warn, warned, warning				mogelijk te voorkomen, is belangrijk<br><br>Het geeft een gerust gevoel als er bij het zorgteam een signaal of 'alarm' afgaat als er een mogelijk risicovolle situatie met een bewoner is	an important role here]<br><br>I feel safe when I don't have to worry about my safety or that of residents / my loved one
			Security (Societal)	Safety and stability in the wider society	National security, social order and stability, stable government,		national security/ nation safe from enemies, social order/societal stability	It is important to him:<br>-that his country is secure and stable<br>-to have a strong state that can defend its citizens<br>-that his country protect itself against all threats	Het is voor hem belangrijk:<br>-dat zijn land veilig en stabiel is<br>-dat het land sterk is en het inwoners kan beschermen<br>--dat zijn land zichzelf kan beschermen tegen alle bedreigingen		
		Tradition	Tradition	Maintaining and preserving cultural, family or religious traditions	Maintaining traditions, doing things in traditional ways	accustomed, Allah, almighty, ancestors, ancestry, angel, angels, atheist, awe, belief, believers, bible, bless, blessed, blessing, blessings, brood, catholic, ceremonies, ceremony, chapel, Christ, Christianity, Christians, church, churches, clan, commandments, congregation, conscience, corrupted, damned, descendants, devil, devout, divinity, ethical, ethics, faith, faithful, folk, folklore, generations, god, grace, habit, habits, heaven, heavenly, heavens, heritage, historical, holiness, holy, honoring, inheritance, innocence, institution, Islam, Jesus, legacy, marriage, marriages, married, missionary, Mohammed, moral, morality, morals, mosque, Muhammad, Muslim, mystic, myth, origins, orthodox, prayer, praying, preaching, priest, prophet, protestant, providence, purity, qualms, relatives, religion, religions, religious, righteousness, rite, ritual, rituals, sacred, savior, scripture, sermon, sermons, service, siblings, sin, soul, theology, tradition, traditional, traditionally, traditions, virtue, worship	respect tradition/preserve customs, devout/hold, religious faith	It is important to him:<br>-to maintain traditional values and ways of thinking<br>-to follow his family's customs or the customs of a religion<br>-to honor the traditional practices of his culture	Het is belangrijk voor hem:<br>-om traditionele waarden en manieren van denken te behouden<br>-om zijn familie gewoonten of religieuze gewoonten na te leven<br>-om de traditionele praktijken van zijn cultuur te eren	*Aan gewoonten vasthouden*<br>Zo doen/deden we het altijd, waarom moet het anders?<br><br>Het is fijner om de bekende manier van werken aan te houden<br><br>Nieuwe zorgtechniek zijn best spannend, we zijn het niet gewend	*Sticking to habits*<br>This is how we always do/did it: why should we change?<br><br>It is better to stick to a familiar way of working<br><br>New things are [/health care technology is] quite exciting because they are not unfamiliar
		Conformity	Conformity (Rules)	Compliance with rules, laws and formal obligations	compliance with expectations, with laws, to rules and authority, being obedient behave properly, avoid doing 'wrong' things	abide, accord, accordance, adequately, appropriate, appropriately, authorities, authorization, authorized, avoid, avoided, avoiding, certainty, code, combination, commitment, commitments, committed, committing, compliance, comply, composure, concession, confines, consensus, consent, conservation, conservative, convention, conventional, conventions, courtesy, covenant, credibility, decency, decree, directions, discipline, duties, duty, entity, establishment, etiquette, familiar, fasting, flexibility, forbidden, formally, hesitate, hierarchy, honestly, humble, humility, identity, imposing, improper, inappropriate, instructed, instruction, integrity, law, laws, legal, legislation, legitimate, mainstream, maintaining, measure, modest, modesty, must, obedience, obey, obligated, obligation, obliged, official, orders, organization, outrageous, patience, periodic, permitting, policies, policy, polite, prescription, preserved, procedure, punishment, reason, reasonable, regularly, regulation, regulations,	self-discipline/resist temptation, obedient/meet obligations	It is important to him:<br>-never to violate rules or regulations<br>-to follow rules even when no-one is watching<br>-to obey all the laws	Het is belangrijk voor hem:<br>-om nooit regels of voorschriften te schenden<br>-om regels te volgen, zelfs als niemand toekijkt<br>-om aan alle wetten te gehoorzamen	*Aan afspraken houden*<br>Wat we samen gekozen hebben om te doen gaat boven mijn eigen mening<br><br>Niet tegen afspraken ingaan<br><br>Aan regels/ afspraken houden<br><br>Doen wat van je verwacht wordt	*Keeping agreements*<br>What we have chosen to do together takes precedence over my own opinion<br><br>Don't go against agreements<br><br>Stick to rules/agreements<br><br>Do what is expected of you
			Conformity (Interpersonal)	Avoidance of upsetting or harming other people	Avoiding to upset others, being polite, show respect to others, honor parents		politeness/ courtesy, honour parents/show respect	It is important to him:<br>-to avoid upsetting other people<br>-never to annoy anyone<br>-never to make other people angry	Het is belangrijk voor hem:<br>-om het van streek maken van anderen te vermijden<br>-om nooit iemand te ergeren<br>-om nooit andere mensen kwaad te maken		

						repetitive, required, requirement, reserve, resignation, resolution, respect, respected, respects, restraint, restricted, restrictions, restrictive, reverence, routinely, rule, rules, ruling, served, shame, shyness, similarity, standards, structure, subordinate, suited, system, trust, trusted, unison, violate, violated, warrant, withholding					

Ponizovskiy, V., Ardag, M., Grogorryan, L, Boyd, R., Dobewall, H., Holtz, P. (2020). Development and validation of the personal values dictionary: A theory driven tool for investigating referenves of the basuc human values in text. *European Journal of Personality*, *34*, 885-902. https://doi.org/10.1002/per.2294

Schwartz, S. H. (2012a). An overview of the Schwartz Theory of Basic Values. *Online readings in psychology and culture*, 20. https://doi.org/dx.doi.org/10.9707/2307-0919.1116

Schwartz, S. H. (2012b). Refining the theory of basic individual values. *Journal of Personality and Social Psychology*. https://doi.org/10.1037/a0029393

Schwartz, S. H. (2021). A Repository of Schwartz Value Scales with Instructions and an Introduction. *Online readings in psychology and culture, International Association for Cross-Cultural Psychology*, *2*, 11. https://doi.org/https://doi.org/10.9707/2307-0919.1173
